# Supplementary material for: Gastric Serotonin Biosynthesis and Its Functional Role in L-Arginine-Induced Gastric Proton Secretion
Source: Int J Mol Sci. 2021 May 30;22(11):5881. doi: 10.3390/ijms22115881 (PMC8199169; doi:10.3390/ijms22115881)
Supplement: Supplementary file 1 [file ijms-22-05881-s001.zip › ijms-1199251-supplementary.pdf]

Supplementary Materials: The following are available online at www.mdpi.com/xxx/s1,

Methods and materials:

Immunofluorescence (HGT-1 cells)

After treatment of HGT-1 cells either with DMEM only or tryptophan hydroxylase inhibitor PCPA, the cells were washed twice with PBS and blocked for 1 h with 5% FBS and 0.5% Triton X-100 in PBS (blocking solution) at room temperature and gentle agitation. Then, the solution was exchanged with primary antibody (1:100, anti-serotonin, raised in rabbit, Merck Millipore) diluted in blocking solution and incubated first at 37°C prior to incubation for 15 min at room temperature with gentle agitation. Thereafter, the cells were washed four times with PBS for five minutes each. Then, secondary antibody (1:1000, Alexa-488, goat anti-rabbit (Molecular Probes, Thermo Fisher Scientific), diluted with PBS containing 5% FBS and 0.2% Triton X-100) was added and incubated for 1 h at room temperature with gentle agitation. Afterwards, the cells were washed four times with PBS for 5 min each. During the last wash, 5 µL of DAPI stain (fixed cells nucBlue, Molecular Probes, Thermo Fisher Scientific) were added. Finally, the coverslip was briefly dipped into ddH2O and mounted using Immu-Mount solution (Life Technologies, Thermo Scientific). Fluorescent images were taken using an EVOS FL microscope (Thermo Fisher Scientific) equipped with an Olympus 10x objective. For control purposes, images of untreated cells, cells treated with primary antibody only and cells treated with secondary antibody only were taken. For testing the specificity of the primary antibody, some coverslips were incubated with primary antibody, which was exposed to pure serotonin for 30 min prior to the experiment. Data analysis was carried out using Image J, assessing the fluorescence of at least 150 cells per image of two technical replicates.

Serotonin ELISA

HGT-1 cells were seeded in 24-well plates at a density of 1.5×10^5 cells per well. One day after seeding, the cells were washed with pre-warmed PBS and subsequently incubated with 200 µL KRHB (supplemented with 0.1% ascorbic acid, pH 7.4) or 30 mM L-Arg for 5 min in a humidified incubator at 37°C in the dark. Since L-Arg induced a change in the pH, both a pH control and a pH adjusted L-Arg solution were tested in addition. The incubation solutions having a pH of 9.5 were passed through a 0.22 µm filter prior to incubation to remove insoluble material. To assess whether 5-HT3 receptors are involved, co-incubations with 10 µM 5-HT3 antagonist granisetron were carried out. After incubation, the supernatant was collected, diluted 1:5 with KRHB and the serotonin concentration assessed using a serotonin-sensitive ELISA (DLD Diagnostika, Hamburg, Germany). Caco-2 cells were seeded in a density of 350 000 cells per well in 12-well plates and differentiated for 21 days, within which period the culture medium was exchanged every two to three days. On day 21, the cells were washed with pre-warmed PBS, incubated for 5 min with 150 µL KRHB (+0.1% ascorbic acid), the supernatant collected and used in the ELISA without further dilution. Similarly, QGP-1 cells were seeded at a density of 250,000 cells per well in 24-well plates, settled for 72 h, and incubated with 250 µL KRHB (+0.1% ascorbic acid) and the collected supernatant centrifuged for 5 min at 1,000x g at 4°C and tested for its serotonin content without further dilution.

LC-MS/MS

The cells were seeded and incubated as described above. However, the collected supernatants were acidified with 0.1% formic acid, and passed through a 0.2 µm filter prior to LC-MS/MS analysis. A total of 50 µL was injected. The analysis was carried out on a Shimadzu LC-MS 8040 equipped with a Synergi 4 µm Fusion-RP 80 A column (150 x 2 mm, Phenomenex) and a security guard cartridge (Phenomenex). A binary gradient of water (+0.1% formic acid, A) and acetonitrile (+0.1% formic acid, B) was used in the chromatographic separation: 0-3 min 0% B, 3-10 min change to 90% B, 13-18 min 90% B, 18-23 min change to 0% B, 23-30 min 0% B. In order to protect the MS from contamination, the eluent was directed to the waste outlet from 0 to 2 min and 8.7 to 30 min, while everything eluting between 2 and 8.7 min was directed to the MS. The MRM settings chosen are shown in Table 2. The MS interface was set as follows: nebulizing gas flow 3 L min-1, DL temperature 150°C, heat block temperature 350°C and drying gas flow 17 L min-1. The calibration range used for the quantification of serotonin in HGT-1 supernatants was 0.4 – 8.3 ng mL-1.

Supplemental Tables

Table S1: Sequence and product size of the primers used for qPCR experiments

Target	Forward primer	Reverse primer	Product size
**TPH1**	TAAGACCTGGGGAACCGTATT	TGGAAAAACCTGTACGCTCTTT	173
**TPH2**	ATCTCGGCGAAGAAGTTCTGA	CAGGGCACATCCTCTAGCTC	165
**AADC**	TGGGGACCACAACATGCTG	TCAGGGCAGATGAATGCACTG	121
**HTR1A**	TCATCGTGGCTCTTGTTCTG	CGGGGTTAAGCAGAGAGTTG	108
**HTR1B**	CTGGTGTGGGTCTTCTCCAT	AGAGGATGTGGTCGGTGTTC	109
**HTR2A**	GTTGCTTACTCGCCGATGATA	TGCCAAGATCACTTACACACAAA	144
**HTR3A**	GAAGCCAACCACCGTATCCAT	CCACATCCACGAACTCATTGAT	218
**HTR3B**	TCTCCCTACCTCTAAGTGCCA	CTCAATGGTCCCAGATGAGTTC	116
**HTR3C**	TTCCGGTCTCACTGCCTATATC	AAGGTGAAGGTACAGTTCTGTTG	129
**HTR3D**	CCCTACGTGGTAAACTTTCTGG	TGTGATGAAGTGCTAGTGGCT	179
**HTR3E**	AGACGCATCCCGGAACATC	GGCACGAGAAGGTTTATGACA	165
**HTR7**	CTCCATCACCTTACCTCCACTC	ATGCCACTGCGGTAGAGTAAAT	110
**SLC6A4**	ACGGAGTTCTACAGAAGGTTGT	ATAGAGTGCCGTGTGTCATCT	118
**TBP**	CCCGAAACGCCGAATATAATCC	GACTGTTCTTCACTCTTGGCTC	130
**GAPDH**	AGGTCGGAGTCAACGGATTTG	GGGGTCATTGATGGCAACAATA	95
**PPIA**	CCACCAGATCATTCCTTCTGTAGC	CTGCAATCCAGCTAGGCATGG	144

Table S2: MRM settings for the simultaneous detection of L-Trp, 5-HTP and serotonin by LC-MS/MS

Target	Transition (m/z)	Dwell time (ms)	Q1 pre bias (V)	CE (V)	Q3 pre bias (V)
**L-Trp**	205 -> 146	400	-12	-19	-13
**5-HTP**	221 -> 204	400	-12	-13	-13
	221 -> 162	400	-12	-19	-13
**5-HT**	177 -> 160	400	-12	-13	-13
	177 -> 115	400	-12	-30	-13

Supplemental Figures

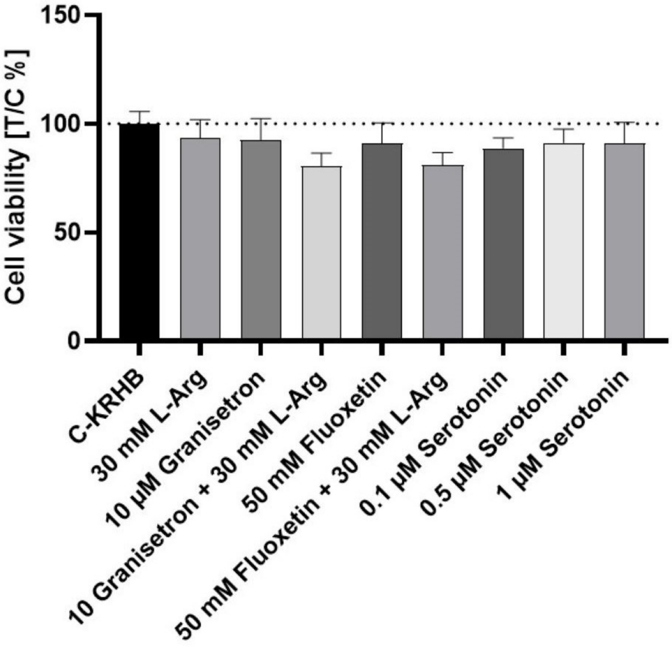

Figure S1 Results of the cell viability assay by means of MTT assay. Data are presented as means ± SD % treated over control (KRHP) n=2-3 with 3 technical replicates.

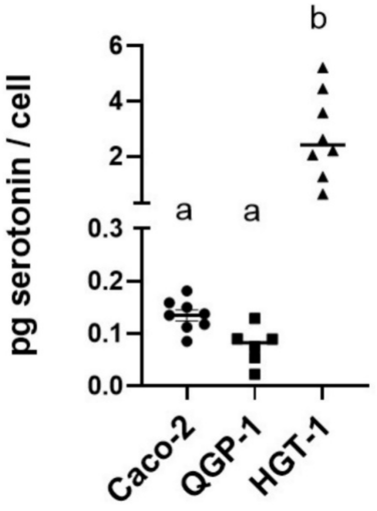

Figure S2 Pg/ serotonin cell n=3-4 with 2 technical replicates each. Statistics: One-Way ANOVA with Holm-Sidak post hoc test. Distinct letters indicate significant difference ($p<0.001$)

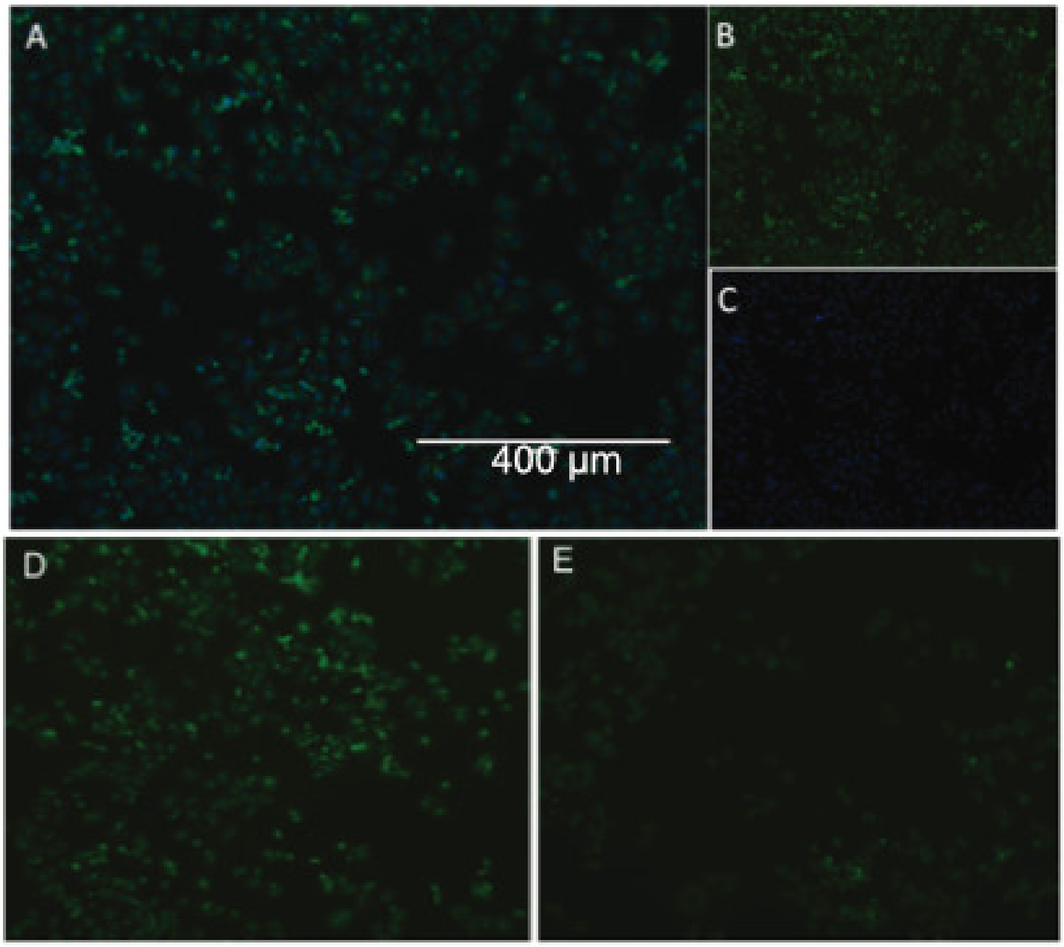

Figure S3 Immunofluorescence of HGT-1 cells. Overlay of nuclear staining and serotonin staining (A), serotonin staining only (B), nuclear staining only (C). A representative image is shown; at least three biological replicates and multiple technical replicates have been stained. Immunofluorescence of HGT-1 cells treated either with DMEM only (D) or tryptophan hydroxylase inhibitor PCPA (E). n=1, tr=2.

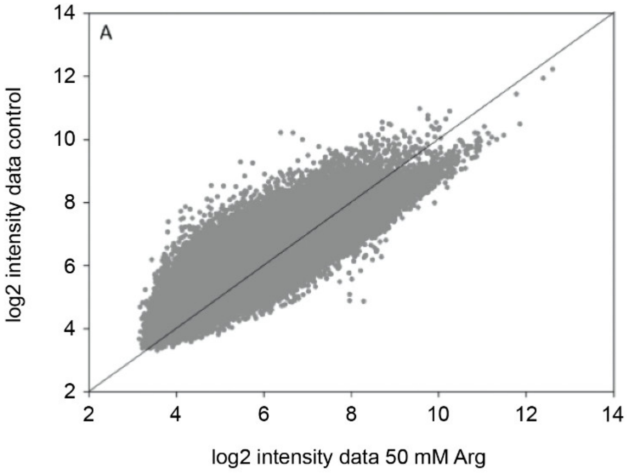

Figure S4 Scatter plot of log2 transformed intensities of control and treatment with L-Arg for 3h. The diagonal line represents a fold change of 1, i.e. equal intensity in control and treatment.

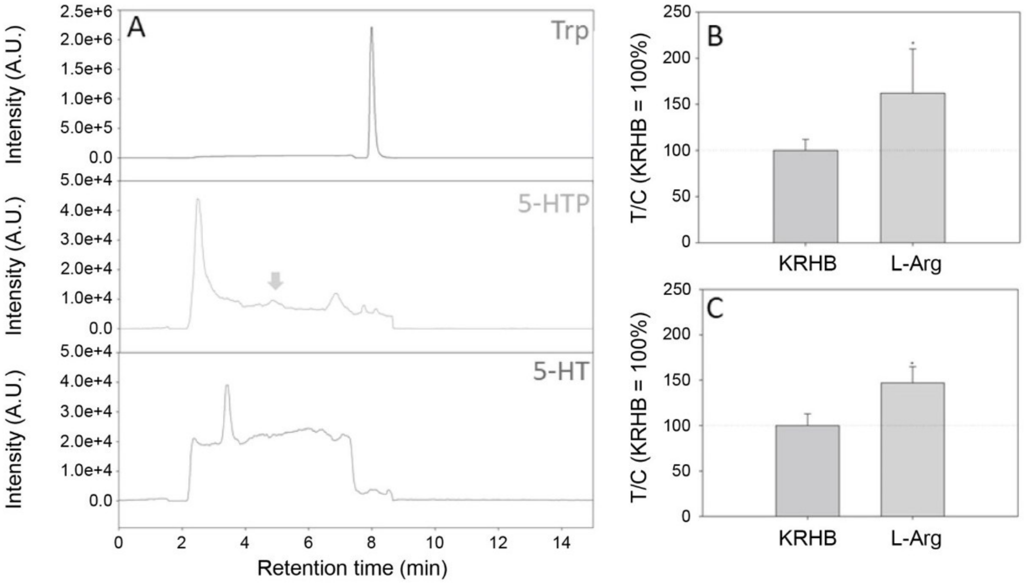

Figure S5 Typical LC-MS/MS chromatogram of the cellular supernatant collected from HGT-1 cells, showing the traces of L-Trp, 5-HTP and 5-HT (A). Without addition of 5-HTP, its retention time is shown with an arrow. Serotonin in the cellular supernatant after treatment of HGT-1 cells with 30 mM L-Arg for 5 min analysed by ELISA (B, n=5) or LC-MS/MS (C, n=4). Data are shown in relation to the control (set to 100%); statistics: Student's t-test (*: $p < 0.05$)

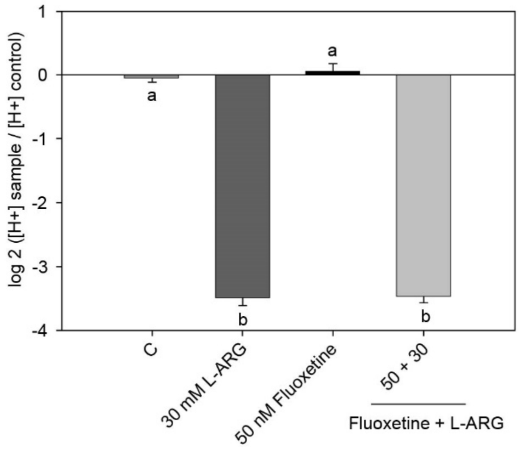

Figure S6 Impact of SERT inhibitor fluoxetine on L-Arg induced proton secretion, n=3, tr= 3-6, Statistics: one-way ANOVA followed by Holm-Sidak post hoc test. Different letters indicating statistical significance ($p<0.05$).
